# Supplementary material for: Ensemble intelligence prediction algorithms and land use scenarios to measure carbon emissions of the Yangtze River Delta: A machine learning model based on Long Short-Term Memory
Source: PLoS One. 2024 Dec 9;19(12):e0311441. doi: 10.1371/journal.pone.0311441 (PMC11627399; doi:10.1371/journal.pone.0311441)
Supplement: S1 Appendix — (PDF) [file pone.0311441.s001.pdf]

S1 Appendix. Descriptions of input and output variables.

Descriptions of input and output variables

Attribute of variables		Variables	Unit	Symbol	Meaning	Source
S1 (Energy consumption stage)	Input	Built-up area	square kilometer	X1	Areas actually developed and built up within the city's administrative districts	China Statistical Yearbook
		Number of employees in industrial enterprises	all the people	X2	Number of persons formally engaged in work, employed in industrial enterprises and projects	China City Statistical Yearbook
		Number of real estate employees	all the people	X3	Refers to the total number of people working in the real estate industry	China City Statistical Yearbook
		Total energy consumption	tons	X4	The sum of all energy sources consumed by the material and non-material production sectors in the country over a given period of time	China Energy Statistical Yearbook
		Real estate development investment completed	$10,000,000	X5	Refers to the amount of investment fully spent on housing construction works, land development works, and investment in public welfare buildings and land acquisition costs, etc.	China City Statistical Yearbook
	Output	Industrial output value	$10,000,000	X6	Final results of industrial production activities of industrial enterprises expressed in monetary terms during the reporting period	China City Statistical Yearbook
		Real estate development enterprises commodity housing sales income	$10,000,000	X7	Revenue from the sale of commercial properties by property development enterprises during the reporting period	China City Statistical Yearbook
		Industrial land area	square kilometer	X8	Area occupied by industrial	China City Statistical Yearbook

					enterprises	
		Residential land area	square kilometer	X9	Total area of residential land, public building land, road and square land and public green space, etc.	China City Statistical Yearbook
		Carbon emissions from industrial land (**not expected**)	tons	X10	Industrial carbon emissions not only have an impact on the environment, but also have a negative impact on the industrial and energy structures	China City Statistical Yearbook
		Carbon emissions from residential land use (**not desired**)	tons	X11	As a high energy consuming and greenhouse gas emitting sector, the real estate industry is one of the larger carbon emitters in the process of urbanization	China City Statistical Yearbook
Link		Comprehensive discharge index of three industrial wastes	tons	X12	Combined emissions of waste gas, wastewater and waste residues from industrial production processes	China Environmental Statistical Yearbook
		Comprehensive discharge index of domestic waste	tons	X13	Integrated discharge of all kinds of solid wastes abandoned by urban life, municipal construction and commercial activities, etc.	China Environmental Statistical Yearbook
**S2 (Sustainable land use stage)**	Input	Urban environmental protection inputs	$10,000,000	X15	Government inputs and support for urban environmental protection projects during the sustainable land use phase	China City Statistical Yearbook
	Output	Harmless treatment of municipal domestic waste	%	X16	The indicator reflects the level of treatment of domestic waste in urban areas	China City Statistical Yearbook
		Urban sewage treatment rate	%	X17	Indicators for measuring the capacity and efficiency of municipal wastewater treatment	China City Statistical Yearbook
		Comprehensive utilisation rate of general industrial solid waste	%	X18	Comprehensive use of industrial solid waste as a percentage of industrial solid waste generation	China City Statistical Yearbook
		Urban green space carbon sink	ton	X19	Processes by which urban green space plants	China City Statistical Yearbook

					absorb atmospheric carbon dioxide and fix it in vegetation and soil, reducing atmospheric carbon dioxide concentrations	
Carry-over		Fixed assets	$10,000,000	X14	Aggregate term for the amount of work involved in the construction and acquisition of fixed assets and the costs associated with it, expressed in monetary terms	China Statistical Yearbook
