# Supplementary material for: Ensemble intelligence prediction algorithms and land use scenarios to measure carbon emissions of the Yangtze River Delta: A machine learning model based on Long Short-Term Memory
Source: PLoS One. 2024 Dec 9;19(12):e0311441. doi: 10.1371/journal.pone.0311441 (PMC11627399; doi:10.1371/journal.pone.0311441)
Supplement: Attachment — Submitted filename: Specific Comments.pdf [file pone.0311441.s004.pdf]

Advantages:1.Significant research topics: Focusing on the carbon emission measurement and prediction in the Yangtze River Delta region is of great practical significance for the study of carbon emission issues in the process of urbanization.

2.Method synthesis: Dynamic two-stage data envelope analysis (DEA) and long-term and short-term memory (LSTM) model are used to comprehensively evaluate the carbon emission efficiency of land use.

3. The literature review is comprehensive: the relevant concepts of carbon emission efficiency of land use are clearly defined, the factors affecting carbon emission efficiency are comprehensively sorted out, and the relevant methods are summarized.

Disadvantages: 1.Insufficient interpretation of the results: in the analysis of the results, the interpretation of some phenomena and conclusions is not deep enough, and there is a lack of discussion on the mechanism behind them. For example, the analysis of the causes for the differences in carbon emission efficiency in different regions is not comprehensive enough, so more in-depth analysis should be conducted from the aspects of economic structure, industrial policy, energy structure and so on, and more targeted suggestions and measures should be put forward. 2. Writing expression needs to be improved: some sentences in the paper are not accurate or fluent, and need to be further modified and improved. For example, avoid using unprofessional expressions such as "we". It is suggested to improve the writing quality and expression accuracy of the paper.

3. Insufficient interpretation of variables: the data source of each variable is not clearly specified in the paper; the large sample problems required by the deep learning algorithm LSTM were not fully considered, the effect size test was not fully conducted, and the concerns about possible overfitting problems were not effectively solved. It is suggested that the variable meaning and data sources of all equations be explained in detail in the paper to enhance the comprehensibility of the paper.
