# Supplementary material for: Ensemble intelligence prediction algorithms and land use scenarios to measure carbon emissions of the Yangtze River Delta: A machine learning model based on Long Short-Term Memory
Source: PLoS One. 2024 Dec 9;19(12):e0311441. doi: 10.1371/journal.pone.0311441 (PMC11627399; doi:10.1371/journal.pone.0311441)
Supplement: Attachment — Submitted filename: 8.5-comments(1).docx [file pone.0311441.s003.docx]

Journal Requirements:

When submitting your revision, we need you to address these additional requirements.

Dear editors and reviewers,

Thank you for giving us the opportunity to submit a revised draft of the manuscript “Ensemble intelligence predictions algorithms and land use scenarios to measure carbon emission of Yangtze River Delta region：A machine learning model based on Long Short-Term Memory” for publication in the Journal of PLOS ONE. We appreciate the time and effort that you and the reviewers dedicated to providing feedback on our manuscript and are grateful for the insightful comments on and valuable improvements to our paper. We have incorporated most of the suggestions made by the reviewers. In the revised manuscript, all the changes are highlighted in yellow for easy inspection. Please see below, for a point-by-point response to the reviewers’ comments and concerns. According to the reviewer’s comments, we have revised the manuscript extensively. If there are any other modifications we could make, we would like very much to modify them and we really appreciate your help.

1. Please ensure that your manuscript meets PLOS ONE's style requirements, including those for file naming. The PLOS ONE style templates can be found at 
https://journals.plos.org/plosone/s/file?id=wjVg/PLOSOne_formatting_sample_main_body.pdf and 
https://journals.plos.org/plosone/s/file?id=ba62/PLOSOne_formatting_sample_title_authors_affiliations.pdf

**Answer：**Thank you for your comments on our article. We have modified the article format to meet the journal requirements.

2. Please note that PLOS ONE has specific guidelines on code sharing for submissions in which author-generated code underpins the findings in the manuscript. In these cases, all author-generated code must be made available without restrictions upon publication of the work. Please review our guidelines at https://journals.plos.org/plosone/s/materials-and-software-sharing#loc-sharing-code and ensure that your code is shared in a way that follows best practice and facilitates reproducibility and reuse.

**Answer：**Thank you for your nice comments on our article. All the codes we use can be shared on the public platform, and we will submit them in the following attachments.

3. We note that the grant information you provided in the ‘Funding Information’ and ‘Financial Disclosure’ sections do not match.

When you resubmit, please ensure that you provide the correct grant numbers for the awards you received for your study in the ‘Funding Information’ section.

**Answer：**Thank you for your nice suggestions on our article. We have provided the correct grant numbers for the awards we received for the study in the‘Funding Information’section.

4. We note that Figure 3 in your submission contain [map/satellite] images which may be copyrighted. All PLOS content is published under the Creative Commons Attribution License (CC BY 4.0), which means that the manuscript, images, and Supporting Information files will be freely available online, and any third party is permitted to access, download, copy, distribute, and use these materials in any way, even commercially, with proper attribution. For these reasons, we cannot publish previously copyrighted maps or satellite images created using proprietary data, such as Google software (Google Maps, Street View, and Earth). For more information, see our copyright guidelines: http://journals.plos.org/plosone/s/licenses-and-copyright.
 
We require you to either (1) present written permission from the copyright holder to publish these figures specifically under the CC BY 4.0 license, or (2) remove the figures from your submission:
 
a. You may seek permission from the original copyright holder of Figure 3 to publish the content specifically under the CC BY 4.0 license.  
 
We recommend that you contact the original copyright holder with the Content Permission Form (http://journals.plos.org/plosone/s/file?id=7c09/content-permission-form.pdf) and the following text:
“I request permission for the open-access journal PLOS ONE to publish XXX under the Creative Commons Attribution License (CCAL) CC BY 4.0 (http://creativecommons.org/licenses/by/4.0/). Please be aware that this license allows unrestricted use and distribution, even commercially, by third parties. Please reply and provide explicit written permission to publish XXX under a CC BY license and complete the attached form.”
 
Please upload the completed Content Permission Form or other proof of granted permissions as an ""Other"" file with your submission.
 
In the figure caption of the copyrighted figure, please include the following text: “Reprinted from [ref] under a CC BY license, with permission from [name of publisher], original copyright [original copyright year].”
 
b. If you are unable to obtain permission from the original copyright holder to publish these figures under the CC BY 4.0 license or if the copyright holder’s requirements are incompatible with the CC BY 4.0 license, please either i) remove the figure or ii) supply a replacement figure that complies with the CC BY 4.0 license. Please check copyright information on all replacement figures and update the figure caption with source information. If applicable, please specify in the figure caption text when a figure is similar but not identical to the original image and is therefore for illustrative purposes only.
The following resources for replacing copyrighted map figures may be helpful:
 
USGS National Map Viewer (public domain): http://viewer.nationalmap.gov/viewer/
The Gateway to Astronaut Photography of Earth (public domain): http://eol.jsc.nasa.gov/sseop/clickmap/
Maps at the CIA (public domain): https://www.cia.gov/library/publications/the-world-factbook/index.html and https://www.cia.gov/library/publications/cia-maps-publications/index.html
NASA Earth Observatory (public domain): http://earthobservatory.nasa.gov/
Landsat: http://landsat.visibleearth.nasa.gov/
USGS EROS (Earth Resources Observatory and Science (EROS) Center) (public domain): http://eros.usgs.gov/#
Natural Earth (public domain): <http://www.naturalearthdata.com>

**Answer：**Thank you for your nice suggestions on our article. We choose to replace the original graph with a new one.

We used images from the open data platform you listed and marked our study area. Here is the link: USGS EROS (Earth Resources Observatory and Science (EROS) Center) (public domain): http://eros.usgs.gov/#
 (Lines 354-356)

Figure 3. Overview of the Study Area.

Note: The images are from the open data platform. USGS EROS (Earth Resources Observatory and Science (EROS) Center) (public domain): http://eros.usgs.gov/#

Reviewer 1#:
Advantages:
1.The literature review is comprehensive and logical, very logical.
2. The content is comprehensive and in-depth depth. Paper on land use of carbon efficiency related concept of the clear definition, the factors of carbon efficiency comprehensive comb, and through the empirical analysis discusses the YRD city cluster in different stages of carbon emission efficiency, including the overall efficiency, stage efficiency, important input and output index analysis and influence factors analysis, etc., detailed and in-depth content.

Dear reviewer,

Thank you for giving us the opportunity to submit a revised draft of the manuscript “Ensemble intelligence predictions algorithms and land use scenarios to measure carbon emission of Yangtze River Delta region：A machine learning model based on Long Short-Term Memory” for publication in the Journal of PLOS ONE. We appreciate the time and effort that you and the reviewers dedicated to providing feedback on our manuscript and are grateful for the insightful comments on and valuable improvements to our paper. According to your nice comments, we have revised the manuscript extensively. If there are any other modifications we could make, we would like very much to modify them and we really appreciate your help.

Disadvantages:
1. There are too many old references, and 3-5 years should account for 55% -60%. It is recommended to increase the literature quoting authoritative journal papers for 3-5 years.

**Answer:** Thank you for your kind comment. We have added the literature quoting authoritative journal papers for 3-5 years. (Lines 869-1004)

[1] Luo, H.J., Zhang, Y.W., Gao, X.Y., Liu, Z.G., Song, X., Meng X.Z., Yang, X.H., Yang, X.H., 2024. Unveiling land use-carbon Nexus: Spatial matrix-enhanced neural network for predicting commercial and residential carbon emissions. Energy. Volume 305. 131722, 0360-5442.
[2] Luo, H.Z., Wang, C.L., Li, C.B., Meng, X.Z., Yang X.H., Tan, Q., 2024. Multi-scale carbon emission characterization and prediction based on land use and interpretable machine learning model: A case study of the Yangtze River Delta Region, China. Applied Energy, Volume 360. 122819, 0306-2619.
[3] Weiss MC, Adusumilli S, Jagai JS, Sargis RM., 2023. Transportation-related environmental mixtures and diabetes prevalence and control in urban/ metropolitan counties in the United States. J Endocr Soc;7(6): bvad062.
[4] Attari MYN, Ala A, Khalilpourshiraz Z., 2022. The electric power supply chain network design and emission reduction policy: a comprehensive review. Environ Sci Pollut es;29:55541–67.
[5] Wang Q, Li L, Li R., 2023. Uncovering the impact of income inequality and population aging on carbon emission efffciency: an empirical analysis of 139 countries. Sci Total Environ. 857:159508.
[6] Liu Q, Song J, Dai T, Shi A, Xu J, Wang E., 2022. Spatio-temporal dynamic evolution of carbon emission intensity and the effectiveness of carbon emission reduction at county level based on nighttime light data. J Clean Prod. 362:132301.
[7] Gorka M, Bezyk Y, Sowka I., 2021. Assessment of GHG interactions in the vicinity of the municipal waste landfill site-case study. Energies. 14(24):8259.
[8] Wang S, Li Y, Li F, Zheng D, Yang J, Yu E., 2023. Spatialization and driving factors of carbon budget at county level in the Yangtze River Delta of China. Environ Sci Pollut Res. 23:289178.
[9] Tan, J., Wang, R., 2021. Research on evaluation and influencing factors of regional ecological efficiency from the perspective of carbon neutrality. J. Environ. Manag. 294, 113030.
[10] Fattah MA, Morshed SR, Morshed SY., 2021. Multi-layer perceptron-Markov chain-based artificial neural network for modelling future land-specific carbon emission pattern and its influences on surface temperature. SN Appl Sci; 3(3):0359.
[11] Kang T, Wang H, He Z, Liu Z, Ren Y, Zhao P., 2023. The effects of urban land use on energy-related CO2 emissions in China. Sci Total Environ. 870:161873.

2. In the analysis of the results, the explanation of some phenomena and conclusions is not deep enough, and the discussion of the mechanism behind them is lacking. It is suggested that the reasons for the difference in carbon emission reduction efficiency in different regions should be analyzed from economic structure, industrial policy, energy structure and other aspects, and more targeted suggestions and measures should be put forward.

**Answer:** Thank you for the comment. We agree with your suggestions and we have merged the paragraph with the results analysis section, as can be seen in the end of section 4. In addition, we have tried our best to summarize the reasons for the difference in carbon emission reduction efficiency in different regions should be analyzed from economic structure, industrial policy, energy structure and other aspects, and more targeted suggestions and measures should be put forward. Hope to get your approval.

For example, (Lines 711-763)

**“4.4 Discussion**

Taking YRD as the study area, two-stage dynamic DEA, Tapio and LSTM model are used to perform measurement and prediction of TCEI in the perspective of land use. The TCEI in each city of YRD during the study interval is fluctuating, but it is generally at a high level. Nevertheless, it is still worthwhile to consider the heterogeneity of various stages and the underlying causes.

In terms of the TCEI of land use calculation, total efficiency has obvious spatial and temporal heterogeneity. It is not unexpected that provincial capitals, metropolitan areas and coastal developed cities show higher TCEI. Economically developed areas often have strong talent accumulation and resource endowment advantages, thus promoting the innovation of production technology and land use. The cumulative effect of land resources and technological innovations in cleaner production allow these cities to reduce carbon emissions while maintaining productivity. In addition, the economically developed areas have better response effect and implementation of energy conservation and emission reduction policies. Specifically, some cities in Anhui and northern Jiangsu have larger increases, while some cities’ TCEIs rose in a fluctuating state. The carbon emission efficiency of land use in cities in southern Jiangsu is moderately high. TCEI decreases in Zhejiang and Shanghai more substantially, suggesting significant potential for improvement in land use and resource allocation in these areas. Carbon emission performance varies across regions and different urbanization stages, demonstrating significant spatial and cyclical disparities.

In terms of stages, the efficiency values of most cities in the initial stage (energy consumption stage) exceeds the second stage (sustainable land utilization stage). In the initial stage, Zhejiang has the lowest total land use carbon emission efficiency among the four regions, but in the second stage, it has the second highest total efficiency value with smaller fluctuations. These improvements are a result of China’s long-term ecological preservation initiatives aimed at enhancing its cities’ green development[43-44]. In addition，among the six major input-output indicators, Jiangsu exhibits the best performance in terms of the indicators relating to carbon source of construction land and urban environmental protection input. This indicates with the advancement of urbanization and the development of industries in the province that the environmental problems in Jiangsu do not match with the final level of economic development[45].

According to the forecast results of TCEI, there is a trend of further optimization and growth of TCEI in the next six years, which indicates that the Yangtze River Delta urban agglomeration in recent years has controlled the expansion of urban built-up areas and promoted the mixed-use development of central urban areas, which has significantly promoted the improvement of carbon emission efficiency. In other words, intensive land use planning model has a positive impact on optimizing land use structure and carbon emission reduction effect[46]. The identification results of influence factors indicate that green space carbon sequestration capacity and population agglomeration effect are the core driving factors of urban land carbon emissions. Based on this, each region should seek suitable land use methods according to its own characteristics, so that the carbon emissions of each region can reach an appropriate level.

From a research perspective, this study takes the comprehensive carbon emission efficiency of land use as the research goal, focusing on the assessment of TCEI at different stages under the macro -level perspective. Land serves as an important carrier for urban economic development, while different types of land assume different functions in regional economic expansion[47-48]. In addition, 27 cities in YRD are taken as case studies for this study. There are many other studies focusing on carbon emissions throughout Asia as well as worldwide[49-50]. Therefore, future research could include other developing countries’ provinces for analysis to show clearer characteristics to help guide planning, management, and decision-making.”

”

3. The writing and expression of the paper is basically clear, but some sentences are not accurate or fluent enough, which need to be further modified and improved.

**Answer:** Thanks for your suggestion. The writing and expression of the paper has been modified and improved by a native English speaker from the USA. And we hope the revised manuscript could be acceptable for you.

4. The formula serial number format is not uniform.

**Answer:** Thank the reviewer for continuing to point out the details. We think this is an excellent suggestion. We have reordered the formula section.

For example, (Lines 259-350)

Reviewer 2#:
In the manuscript titled “Ensemble intelligence predictions algorithms and land use scenarios to measure carbon emission of Yangtze River Delta region：A machine learning model based on Long Short-Term Memory” , authors performed two-stage dynamic data envelopment analysis and LSTM models and demonstrated regional heterogeneity of carbon emission efficiency. The article is an interesting analysis and prediction of carbon emission efficiency in Yangtze River Delta urban agglomeration. This study contains some interesting findings and are valuable for the understanding of carbon reduction in urban agglomeration. However, lack of updated literature and scholarly language are the major flaw of the study. Therefore, MAJOR revision has to be done before this manuscript could be accepted for publication in the PLOS ONE.

1. The Abstract should explain the reasons for selecting cities in the Yangtze River Delta region as research subjects. (Lines 12-34)

**Answer:** Thank the reviewer for continuing to point out the details. We think this is an excellent suggestion. We have added the reasons for selecting cities in the Yangtze River Delta region as research subjects in the Abstract.

“To enrich case studies for densely populated cities and highly commercialized regions, this research evaluates the total-factor carbon emission efficiency index (TCEI) of 27 cities in China's Yangtze River Delta (YRD) urban agglomeration at different stages from 2011 to 2020 using two-stage dynamic data envelopment analysis (DEA).”

And you can see more details in 1 Introduction section:

In the choice of research objects, the YRD city cluster is the most economically efficient and urban agglomerated city area in China, accounting for 20% of China's economic output despite occupying only 2.1% of its land area. In addition, the total permanent population of YRD in 2023 is 236.9 million people, reflecting the population agglomeration effect of the YRD region. Considering the lack of case studies specifically aimed at densely populated cities and highly commercialized areas, and the need for empirical application of demonstrative methods, this paper chooses the YRD as a case to study the path of energy conservation and emission reduction of urban agglomeration with good economic endowment in developing countries. **‌**

2. Abbreviation should be presented in a table. In addition, please explain their meanings when abbreviations first appear in the main text.

**Answer:** Thank the reviewer for continuing to point out the details. We think this is an excellent suggestion. We have presented Abbreviation in a table and explained their meanings when abbreviations first appear in the main text, as you can see in the second page. (Lines 37-38)

**Abbreviation:**

| IPCC | Intergovernmental Panel on Climate Change |
| --- | --- |
| TCEI | Total-factor carbon emission efficiency index |
| YRD | Yangtze River Delta |
| SBM | Slacks-based measure |
| DMU | Decision-making unit |
| LMDI | Logarithmic mean divisia index |
| DEA | Data envelopment analysis |
| SFA | Stochastic frontier model |
| LSTM | Long Short-Term Memory |
| RNN | Recurrent neural network |
| MPI | Malmquist productivity index |
| St. dev. | Standard deviation |
| CNY | Chinese yuan |
| AVE | Average |
| MSE | Mean-square error |
| MAE | Mean absolute error |

3. It is recommended to add relevant literature from the past three years.

**Answer:** Thank you for your kind comment. We have added the literature quoting authoritative journal papers from the past three years. (Lines 869-1004)

[1] Luo, H.J., Zhang, Y.W., Gao, X.Y., Liu, Z.G., Song, X., Meng X.Z., Yang, X.H., Yang, X.H., 2024. Unveiling land use-carbon Nexus: Spatial matrix-enhanced neural network for predicting commercial and residential carbon emissions. Energy. Volume 305. 131722, 0360-5442.
[2] Luo, H.Z., Wang, C.L., Li, C.B., Meng, X.Z., Yang X.H., Tan, Q., 2024. Multi-scale carbon emission characterization and prediction based on land use and interpretable machine learning model: A case study of the Yangtze River Delta Region, China. Applied Energy, Volume 360. 122819, 0306-2619.
[3] Weiss MC, Adusumilli S, Jagai JS, Sargis RM., 2023. Transportation-related environmental mixtures and diabetes prevalence and control in urban/ metropolitan counties in the United States. J Endocr Soc;7(6): bvad062.
[4] Attari MYN, Ala A, Khalilpourshiraz Z., 2022. The electric power supply chain network design and emission reduction policy: a comprehensive review. Environ Sci Pollut es;29:55541–67.
[5] Wang Q, Li L, Li R., 2023. Uncovering the impact of income inequality and population aging on carbon emission efffciency: an empirical analysis of 139 countries. Sci Total Environ. 857:159508.
[6] Liu Q, Song J, Dai T, Shi A, Xu J, Wang E., 2022. Spatio-temporal dynamic evolution of carbon emission intensity and the effectiveness of carbon emission reduction at county level based on nighttime light data. J Clean Prod. 362:132301.
[7] Gorka M, Bezyk Y, Sowka I., 2021. Assessment of GHG interactions in the vicinity of the municipal waste landfill site-case study. Energies. 14(24):8259.
[8] Wang S, Li Y, Li F, Zheng D, Yang J, Yu E., 2023. Spatialization and driving factors of carbon budget at county level in the Yangtze River Delta of China. Environ Sci Pollut Res. 23:289178.
[9] Tan, J., Wang, R., 2021. Research on evaluation and influencing factors of regional ecological efficiency from the perspective of carbon neutrality. J. Environ. Manag. 294, 113030.
[10] Fattah MA, Morshed SR, Morshed SY., 2021. Multi-layer perceptron-Markov chain-based artificial neural network for modelling future land-specific carbon emission pattern and its influences on surface temperature. SN Appl Sci; 3(3):0359.
[11] Kang T, Wang H, He Z, Liu Z, Ren Y, Zhao P., 2023. The effects of urban land use on energy-related CO2 emissions in China. Sci Total Environ. 870:161873.

4. The research gap should be listed in sections after the Literature Review.

**Answer：**Thank you for your insightful comment and kind suggestion. We agree with the reviewer’s assessment. Therefore, we have tried our best to summarize the research gap in the end of the Literature Review.

(Lines 207-223)

“In summary, some shortcomings are also found from other studies' results. First, the majority of the studies have been focused on the accounting for TCEI of specific land use types, thereby overlooking macro-level land use efficiency measurement. This also results in a decline in the number of analyses of the driving factors of the overall land use TCEI of a region. Second, in many studies, the effects of non-expected variables and carry-over variables are ignored in the process of calculating TCEI value by DEA, which results in the calculation results cannot objectively reflect the dynamic characteristics of land use carbon emissions. Third, the dynamic two-stage DEA model can be used to assess the whole-factor carbon emission efficiency of different land resource allocation levels at the level of urban agglomerations through a more scientific manner. Ultimately, most studies have primarily concentrated on determining TCEI of land use and lacked a comprehensive forecast of future efficiency values for urban conglomerations. Therefore, this paper presents the two-stage dynamic DEA-Tobit-LSTM model in the realm of artificial intelligence that can efficiently interprets the forecast results by contrasting the outcomes of dynamic scenario simulation using the deep learning approach. This supplements the body of literature related to the prediction of trends in land use carbon emissions.”

5. The Policy Implications are too general, and the feasibility and operability of the policy implications should be improved.

**Answer:** Thank you for your insightful suggestion. We tried our best to improve the manuscript and made some changes to the paper. We tried to optimize the conclusion part and put forward more targeted suggestions.

For example, (Lines 817-854)

“5.3 Suggestions

Each region in YRD displays considerable variation in carbon emission efficiency, and there is a notable disparity in the land-use carbon emission performance of each city between the two phases. For this reason, cities with lower carbon emission efficiency should introduce advanced emission reduction and energy-saving technologies and management methods, focus on the green economy industrial belt with a high growth rate, and reduce regional efficiency differences. If the YRD wants to keep the trend of decreasing carbon emission and increasing TECI in the process of land use, it must control the development and construction of construction land. In addition, the main way to reduce carbon emissions from carbon sources is to reduce industrial land and land area of high-energy enterprises, which requires YRD to concentrate on planning industrial parks and large buildings, so as to further achieve its carbon emission reduction target.”

6. In the Introduction section, this article adopts the form of a question to explain the research purpose but fails to clearly explain the contribution of this article.

**Answer:** Thank the reviewer for continuing to point out the details. We think this is an excellent suggestion. We have revised the contribution of this article in the end of 1 Introduction section and divided this part into four aspects.

For example, (Lines 86-100)

“This paper’s innovations and major contributions can be distilled into four main points. First of all, unlike studies that measured carbon emission efficiency through a single dimension of energy consumption, this study takes land use in urban agglomerations as a new entry point, and further optimize the urban carbon emission efficiency index system. In addition, the dynamic two-stage DEA model resolves the issue of subjective assignment of specific production functions in the traditional DEA model and broadens the notion of TCEI measurement. In the identification of influencing factors, this paper quantifies the direction and degree of influence of industrial structure, urban population density, and urban environmental governance level on TCEI, in order to help the government to identify pollution sources and to provide a basis for exploring the sustainable use of land in urban agglomerations and achieving the dual-carbon goal. Lastly, at the level of empirical methods, this study combines the data envelopment analysis approach with the neural network model from the field of artificial intelligence, thus expanding the modeling approaches taken for predicting carbon emission efficiency. It offers a certain degree of generalizability.”

7. In the Research Method section, it was noted that the author did not italicize some variables. Please correct this.

**Answer:** Thank you for your insightful suggestion. We tried our best to improve the manuscript and made some changes to the paper. We tried to italicize some variables in the Research Method section.

For example, (Lines 225-235)

“**3.1 Dynamic two-stage DEA approach**

This study aims to address the issues arising from static analyses and regional disparities by segmenting the approach into two stages: the first (S1) is the energy consumption stage, and the second (S2) is the sustainable land use stage. Assuming that there are *n DMUs* *(j = 1, ..., n)* and the *DMUs* are 27 cities of YRD in this research, each with *k* partitions *(k = 1, ..., k)* and *n* time periods *(T = 1, ..., T)* and the time series is 2011-2020 in this paper, each *DMU* has an input and an output in time period *T* and a carry-over (link) in the next time period *T + 1* to the next time period *(j = 1, ..., n)*. The inputs and outputs of each partition *K* by *K (K, h)i* denote the divisions from *k* to *h*, and *n* and *Lhk* denote the set of divisions from *k* and *h*. We show the inputs and outputs, links, and carry-over definitions as follows.”

8. Method limitations should be briefly covered in the Research Method section

**Answer:** Thank the reviewer for continuing to point out the details. We think this is an excellent suggestion. We have added method limitations sections in the Research Method section.

For example, (Lines 129-132)

“Nevertheless, the development of economic models, like LMDI and STIRPAT, frequently requires the application of an assortment of assumptions and is subject to limitations. The characteristics of data volatility aren't sufficiently explained by econometric models.”

“SFA often requires the establishment of an ideal f-value, and this excessively stringent assumption may result in structural prejudice as a result of an incorrect manufacturing procedure setting[15,16]. Conversely, DEA has been widely employed in the field of carbon emission performance because it directly calculates data without the necessity of pre-estimating parameters [17]. The call for urban development to reduce energy consumption and associated carbon emissions has become more prevalent over the past decade as a result of the pressure to return to green and sustainable development. Furthermore, the conventional DEA model is frequently less precise than the two-stage DEA model with carry-over variables.”

9. There are still some formatting issues in this article, please ask the author to correct them. For example, “Table 2. Changes in the efficiency of energy consumption stages by provinces in the YRD”

**Answer:** Thank the reviewer for continuing to point out the details. We think this is an excellent suggestion. We have modified all the formatting issues in the article.

For example, (Lines 452-455)

Table 2. Changes in the efficiency of energy consumption stages by provinces in the YRD region, 2011-2020.

| **Province** | **Mean** | **2011** | **2012** | **2013** | **2014** | **2015** | **2016** | **2017** | **2018** | **2019** | **2020** |
| --- | --- | --- | --- | --- | --- | --- | --- | --- | --- | --- | --- |
| **Shanghai** | 0.7218 | 1.0000 | 0.6292 | 0.3297 | 0.5613 | 0.3835 | 1.0000 | 1.0000 | 0.9718 | 0.6642 | 0.6779 |
| **Anhui** | 0.7614 | 0.6744 | 0.7073 | 0.6683 | 0.6786 | 0.7014 | 0.8230 | 0.8740 | 0.9003 | 0.7754 | 0.8108 |
| **Jiangsu** | 0.8352 | 0.7072 | 0.6957 | 0.8201 | 0.7340 | 0.7533 | 0.8827 | 0.9593 | 0.9743 | 0.9093 | 0.9166 |
| **Zhejiang** | 0.5695 | 0.5176 | 0.4611 | 0.5599 | 0.4782 | 0.4480 | 0.6403 | 0.7187 | 0.7324 | 0.5719 | 0.5668 |

10. In the Descriptive Statistics section, it is evident that the data in Figures 4 and 5 should not be presented as bar charts, as the minimum values are almost imperceptible. Suggest using a box diagram instead.

**Answer:** Thank the reviewer for continuing to point out the details. We think this is an excellent suggestion. We have replaced bar charts with box diagram to overcome problems that the minimum values are almost imperceptible and added more explanation, which can be seen in Figure 3 and Figure 4. (Lines 397-411)

11. Please do not use such kind of terms, which mean nothing in terms of scientific writing. There are other terms to be corrected too. For example, “we”.

**Answer:** Thank you very much for the highly valuable comment. We have tried to change “We” to “This research”, " Meanwhile " to “In addition” or “Moreover” in order to be more professional, as seen in Abstract section.

For example,

“To enrich case studies for densely populated cities and highly commercialized regions, **this research** evaluates the total-factor carbon emission efficiency index (TCEI) of 27 cities in China's Yangtze River Delta (YRD) urban agglomeration at different stages from 2011 to 2020 using two-stage dynamic data envelopment analysis (DEA).”

“**In addition**，among the six major input-output indicators, Jiangsu exhibits the best performance in terms of the indicators relating to carbon source of construction land and urban environmental protection input. This indicates with the advancement of urbanization and the development of industries in the province that the environmental problems in Jiangsu do not match with the final level of economic development.”

12. There was no mention of the limitation of the study, please add the section.

**Answer:** Thank the reviewer for continuing to point out the details. We think this is an excellent suggestion. We have added the 5.2 Limitations section in 5 Conclusions and implications part, as you can see below.

(Lines 803-816)

**“5.2 Limitations**

The panel data of YRD from 2011 to 2020 was employed to analyze the LUEE and driving factors in this study. The most recent year is currently unavailable due to the time gap in obtaining statistical data. Simultaneously, future research may endeavor to establish a more comprehensive TCEI indicator system and increase the sample size by incorporating indicators that reflect the quality of life of residents and industrial upgrading factors to investigate additional factors that influence environmental efficiency in the context of urbanization.

Moving forward, it is important to investigate the effects of various land types on urban carbon emission efficiency using a macro-micro integrated approach. This will enhance our understanding of the role and influence mechanism of land use efficiency and the entire ecosystem. The goal is to strengthen the symbiotic relationship between the healthy operation of ecosystems and the superior growth of the macro-economy.”

13. The Discussion part is needed, but it should be placed in the result analysis section.
**Answer:** Thank you for the comment. We agree with your suggestions and we have merged the paragraph with the results analysis section, as can be seen in the end of section 4.

(Lines 711-763)

**“4.4 Discussion**

Taking YRD as the study area, two-stage dynamic DEA, Tapio and LSTM model are used to perform measurement and prediction of TCEI in the perspective of land use. The TCEI in each city of YRD during the study interval is fluctuating, but it is generally at a high level. Nevertheless, it is still worthwhile to consider the heterogeneity of various stages and the underlying causes.

In terms of the TCEI of land use calculation, total efficiency has obvious spatial and temporal heterogeneity. It is not unexpected that provincial capitals, metropolitan areas and coastal developed cities show higher TCEI. Economically developed areas often have strong talent accumulation and resource endowment advantages, thus promoting the innovation of production technology and land use. The cumulative effect of land resources and technological innovations in cleaner production allow these cities to reduce carbon emissions while maintaining productivity. In addition, the economically developed areas have better response effect and implementation of energy conservation and emission reduction policies. Specifically, some cities in Anhui and northern Jiangsu have larger increases, while some cities’ TCEIs rose in a fluctuating state. The carbon emission efficiency of land use in cities in southern Jiangsu is moderately high. TCEI decreases in Zhejiang and Shanghai more substantially, suggesting significant potential for improvement in land use and resource allocation in these areas. Carbon emission performance varies across regions and different urbanization stages, demonstrating significant spatial and cyclical disparities.”

Reviewer 3#:
The manuscript entitled Ensemble intelligence predictions algorithms and land use scenarios to measure carbon emission of Yangtze River Delta region：A machine learning model based on Long Short-Term Memory has been evaluated. The study is meaningful, however, the draft needs Major Revisions, please see my comments:

1. Too long a title, and avoid using abbreviations in Abstract.

**Answer:** Thank you for your kind comment. As suggested by the reviewer, the authors have tried to simplify the title of the article to highlight the research focus.

For example,

“Measurements and predictions of land use-carbon emission efficiency via two-stage dynamic DEA-Tobit-LSTM model：a case study of Yangtze River Delta region, China”

2. For too long Abstract, authors need to succinctly describe the research work and report the main results.

**Answer:** Thank you for your nice suggestions. We have tried to simplify the title of the article to briefly describe the research work and report the main results.

For example, (Lines 12-34)

**“Abstract**

In urban agglomerations, land use is the main source of carbon emissions, and reducing carbon emissions and improving land use efficiency are the keys to achieving sustainable development goals. To enrich case studies for densely populated cities and highly commercialized regions, this research evaluates the total-factor carbon emission efficiency index (TCEI) of 27 cities in China's Yangtze River Delta (YRD) urban agglomeration at different stages from 2011 to 2020 using two-stage dynamic data envelopment analysis (DEA). Based on this, the study carries out regression analysis and a long-short-term memory model (LSTM) to filter out the factors and predict TCEI, respectively. The results indicate the following. (1) The total efficiency of 27 cities has significantly improved from 2011 to 2020, and there are obvious spatial heterogeneity characteristics. (2) In terms of stages, most cities' efficiency values in the initial stage (energy consumption stage) exceed those in the second stage (sustainable land utilization stage). (3) In terms of influencing factors, urban green space's ability to capture carbon has a notably positive correlation with carbon emission efficiency. In contrast, the substantial carbon emissions resulting from human respiration are a negative factor affecting carbon emission efficiency. (4) Over the forthcoming six years, the anticipated efficiency value of land use TCEI in the urban cluster of YRD is expected to range between 0.65 and 0.75. Those cities with the highest performance are projected to achieve an efficiency value of 0.9480. Lastly, this research investigates the interaction between actors and land resources on TCEI, resulting in a beneficial understanding for actors to make strategic adjustments during the urbanization process.”

3. The research motivation is not clear, the author should gradually advance to the specific field of research from the perspective of global sustainable development.

**Answer:** Thank you for your constructive feedback. We highly agree with your comments. In fact, our core goal is to achieve the sustainable and green development goals of the Yangtze River Delta city cluster. Based on this, we added some relevant concept to the article.

For example,

“Land use is the main source of carbon emissions in urban agglomerations, and reducing carbon emissions and improving the efficiency of land use are the keys to achieving sustainable development goals. To enrich case studies for densely populated cities and highly commercialized regions, this research evaluates the total-factor carbon emission efficiency index (TCEI) of 27 cities in China's Yangtze River Delta (YRD) urban agglomeration at different stages from 2011 to 2020 using two-stage dynamic data envelopment analysis (DEA). By calculating and predicting the TCEI of land use, it provides valuable insights for actors to carry out energy and industrial restructuring in social-ecological systems, enhance land-use efficiency, and facilitate the balanced and symbiotic progress of humanity and the natural world.”

4. The literature review is inadequate, and emerging research on land use carbon emissions needs to be considered. I will provide representative papers for your reference: <https://doi.org/10.1016/j.energy.2024.131722；https://doi.org/10.1016/j.apenergy.2024.122819；https://doi.org/10.1016/j.jclepro.2023.140069；https://doi.org/10.1016/j.apenergy.2023.121488>

**Answer:** Thank you for your kind comment. We carefully studied the high-quality literature you provided, and got a lot of inspiration and harvest. We are grateful for your help and support. We have added the literature quoting authoritative journal papers for 3-5 years based on the literature you gave. (Lines 869-1004)

[1] Luo, H.J., Zhang, Y.W., Gao, X.Y., Liu, Z.G., Song, X., Meng X.Z., Yang, X.H., Yang, X.H., 2024. Unveiling land use-carbon Nexus: Spatial matrix-enhanced neural network for predicting commercial and residential carbon emissions. Energy. Volume 305. 131722, 0360-5442.
[2] Luo, H.Z., Wang, C.L., Li, C.B., Meng, X.Z., Yang X.H., Tan, Q., 2024. Multi-scale carbon emission characterization and prediction based on land use and interpretable machine learning model: A case study of the Yangtze River Delta Region, China. Applied Energy, Volume 360. 122819, 0306-2619.
[3] Weiss MC, Adusumilli S, Jagai JS, Sargis RM., 2023. Transportation-related environmental mixtures and diabetes prevalence and control in urban/ metropolitan counties in the United States. J Endocr Soc;7(6): bvad062.
[4] Attari MYN, Ala A, Khalilpourshiraz Z., 2022. The electric power supply chain network design and emission reduction policy: a comprehensive review. Environ Sci Pollut es;29:55541–67.
[5] Wang Q, Li L, Li R., 2023. Uncovering the impact of income inequality and population aging on carbon emission efffciency: an empirical analysis of 139 countries. Sci Total Environ. 857:159508.
[6] Liu Q, Song J, Dai T, Shi A, Xu J, Wang E., 2022. Spatio-temporal dynamic evolution of carbon emission intensity and the effectiveness of carbon emission reduction at county level based on nighttime light data. J Clean Prod. 362:132301.
[7] Gorka M, Bezyk Y, Sowka I., 2021. Assessment of GHG interactions in the vicinity of the municipal waste landfill site-case study. Energies. 14(24):8259.
[8] Wang S, Li Y, Li F, Zheng D, Yang J, Yu E., 2023. Spatialization and driving factors of carbon budget at county level in the Yangtze River Delta of China. Environ Sci Pollut Res. 23:289178.
[9] Tan, J., Wang, R., 2021. Research on evaluation and influencing factors of regional ecological efficiency from the perspective of carbon neutrality. J. Environ. Manag. 294, 113030.
[10] Fattah MA, Morshed SR, Morshed SY., 2021. Multi-layer perceptron-Markov chain-based artificial neural network for modelling future land-specific carbon emission pattern and its influences on surface temperature. SN Appl Sci; 3(3):0359.
[11] Kang T, Wang H, He Z, Liu Z, Ren Y, Zhao P., 2023. The effects of urban land use on energy-related CO2 emissions in China. Sci Total Environ. 870:161873.

5. Variables need to be in italics. In addition, the transfer of variables in the numerical model needs to be demonstrated with a clear system diagram.

**Answer:** Thank you for your insightful suggestion. We tried our best to improve the manuscript and made some changes to the paper. We tried to italicize some variables in the Research Method section.

For example, (Lines 225-256)

“**3.1 Dynamic two-stage DEA approach**

This study aims to address the issues arising from static analyses and regional disparities by segmenting the approach into two stages: the first (S1) is the energy consumption stage, and the second (S2) is the sustainable land use stage. Assuming that there are *n DMUs* *(j = 1, ..., n)* and the *DMUs* are 27 cities of YRD in this research, each with *k* partitions *(k = 1, ..., k)* and *n* time periods *(T = 1, ..., T)* and the time series is 2011-2020 in this paper, each *DMU* has an input and an output in time period *T* and a carry-over (link) in the next time period *T + 1* to the next time period *(j = 1, ..., n)*. The inputs and outputs of each partition *K* by *K (K, h)i* denote the divisions from *k* to *h*, and *n* and *Lhk* denote the set of divisions from *k* and *h*. We show the inputs and outputs, links, and carry-over definitions as follows.”

In addition, we have increased the clarity of the image with Visio software, so you can zoom in or out to see the details yourself. As can be seen in “Figure 1. TCEI Assessment Framework.” (Lines 292-294)

6. What are the variables of all the equations respectively, the author needs to explain. Note that each variable corresponds to the data source studied in this paper is the focus, rather than just describing the general name of the variable. For example, t corresponds to time, and the time series is 20XX-20XX in this paper.

**Answer:** Thank you for your insightful comment. We take your question of the variables of all the equations very seriously. We tried our best to improve the manuscript and made some changes to the paper. We tried to explain some variables in the 3 Research Method section and annotate the time range of the selected variables in the 4.1 Data overview section.

For example, (Lines 359-361), (Lines 225-235)

“The data are sourced from the China Statistical Yearbook (2011-2020), China Urban Statistical Yearbook (2011-2020), and China Energy Statistical Yearbook (2011-2020).”

“**3.1 Dynamic two-stage DEA approach**

This study aims to address the issues arising from static analyses and regional disparities by segmenting the approach into two stages: the first (S1) is the energy consumption stage, and the second (S2) is the sustainable land use stage. Assuming that there are *n DMUs* *(j = 1, ..., n)* and the *DMUs* are 27 cities of YRD in this research, each with *k* partitions *(k = 1, ..., k)* and *n* time periods *(T = 1, ..., T)* and the time series is 2011-2020 in this paper, each *DMU* has an input and an output in time period *T* and a carry-over (link) in the next time period *T + 1* to the next time period *(j = 1, ..., n)*.

7. What are the sources of land use data? To my knowledge, the vast majority of land use datasets fail to identify residential and industrial land from built-up areas.

**Answer:** We thank the reviewer for the highly valuable comment. Our land use data is obtained on the open data platform of the Chinese housing and construction sector. You can check the link below for confirmation. In addition, details regarding the area of land used for housing and industrial use are in Section 2.2 of the table.

<https://www.mohurd.gov.cn/gongkai/fdzdgknr/sjfb/tjxx/jstjnj/index.html>.

For example,

2-2 National Urban Population and Construction Land by City （2021）

8. Descriptive statistics are not enough. In addition, LSTM, a deep learning algorithm, requires a large sample size. Has the author conducted an effect size test? In addition, I worry about the emergence of overfitting.

**Answer:** Thank the reviewer for continuing to point out the details. We think this is an excellent suggestion. We have replaced bar charts with box diagram to overcome problems that the minimum values are almost imperceptible and added more explanation, which can be seen in Figure 3 and Figure 4. (Lines 397-411).

In addition, thank you for continuing to point out the details. We think this is an excellent suggestion. Due to the limited sample size, we put a lot of effort into addressing your concerns about the fit during the writing phase and made several optimization adjustments to the number of samples allocated in the training set and the test set. In the end, we started by randomly selecting several of the 27 cities as the training and test sets, and found that this did not occur a complete fit of the model. Specifically, the adjusted R^2^ value of the training set and test set is within the range of 0.06.

We believe that with algorithmic optimization, more variable sample, and more time periods in which data can be obtained, the limitation on sample size will be well addressed. Based on our evaluation, we think the forecast results can make a new discovery: that there may be other factors affecting cities that we need to do more comprehensive research on, which are still worth pondering.

To complete our new attempt to explore the efficiency value prediction, we also made LSTM model forecasts for each input-output index in the next six years (Figure 10. Plot of Input Variable Fit.). According to the results, all the input-output indicators show good fitting effect, and there is no overfitting phenomenon, and the fitting effect has a certain generalization ability, which provides a guarantee for the reliability of the prediction.

9. Calculating specific numbers without explaining why would call into question the contribution and significance of the manuscript.

**Answer:** Thank you for your insightful comment. We take your question of article structure very seriously. Based on your suggestions, we have made significant adjustments to 4.4 Discussion and 5.1 Conclusions to call into question the contribution and significance of the manuscript. And the original conclusion part has been deleted and optimized.

After reading relevant literature, we have read and studied a lot of relevant literature and made the following adjustments to the article structure: (Lines 711-802)

**“5.1 Conclusions**

In this research, we employ a dynamic two-stage DEA model to build a TCEI assessment framework. Subsequently, we determine TCEI in the land use process of YRD from 2011 to 2020 and further predict their dynamic evolution. The findings suggest the following.

1. This study takes land use in the urban carbon emission efficiency index system as a new entry point. Looking at the total efficiency values, the average for the 27 cities during the study period is around 0.6, showing notable differences between groups. Specifically, Anhui has the highest carbon emission efficiency, followed by Jiangsu Province. Zhejiang and Shanghai have the lowest levels. During the study period, TCEI of the YRD city group generally shows an increasing trend within the study interval. This fills the first distribution identified in the 1 Introduction chapter.

2. In terms of phases, during the study period, the efficiency value of the first phase for Shanghai maintained a high level of around 0.7. However, the second phase shows a significant fluctuation in land-use carbon emission efficiency, with a difference exceeding 0.75 between the highest and lowest efficiency values. This suggests that this type of highly developed economic region should take advantage of its own strengths to promote innovative research and develop energy technologies and emission reduction technologies. This phased DEA method overcomes the defects that the traditional DEA model cannot reflect and has higher accuracy and flexibility and indicates the second distribution identified in the 1 Introduction section.

3. In terms of sub-indicators, the contribution of Shanghai to the built-up area indicator is larger. It shows that Shanghai mainly improves the level of urban development by increasing the area of built-up land. As a result, it is crucial to optimize the industrial structure and land resource allocation, and to increase the environmental protection input in areas with inefficient land use and carbon emission. These findings make up for the shortcomings of other DEA models found in the 2 Literature review.

4. From the results of driver identification of green space area and general industrial solid waste emissions on the efficiency of land use carbon emissions are all dominated by positive effects, while the population respiratory carbon emissions and the amount of domestic waste removal are dominated by negative effects. This illustrates the third distribution identified in the 1 Introduction section.

5. From the prediction results, this study selects the LSTM model and predicts that the land use TCEI of YRD will fluctuate in the range of 0.3-1.1 in the next six years. To some extent, it is better than the calculated results of the efficiency values of the two stages. Most of the sample's predicted values fluctuate slightly higher than the efficiency value. This study attempts to make future prediction research based on carbon emission efficiency, reflecting the contribution to the fourth aspect.”
