# Supplementary material for: Ensemble intelligence prediction algorithms and land use scenarios to measure carbon emissions of the Yangtze River Delta: A machine learning model based on Long Short-Term Memory
Source: PLoS One. 2024 Dec 9;19(12):e0311441. doi: 10.1371/journal.pone.0311441 (PMC11627399; doi:10.1371/journal.pone.0311441)
Supplement: Attachment — Submitted filename: 9.3-comments.docx [file pone.0311441.s005.docx]

**Reviewer 1#:**

Dear reviewer,

Thank you for giving us the opportunity to submit a revised draft of the manuscript “Ensemble intelligence prediction algorithms and land use scenarios to measure carbon emissions of the Yangtze River Delta: A machine learning model based on Long Short-Term Memory” for publication in the Journal of PLOS ONE. We appreciate the time and effort that you and the reviewers dedicated to providing feedback on our manuscript and are grateful for the insightful comments on and valuable improvements to our paper. We have incorporated most of the suggestions made by the reviewers. In the revised manuscript, all the changes are highlighted in yellow for easy inspection. Please see below, for a point-by-point response to the reviewers’ comments and concerns. According to the reviewer’s comments, we have revised the manuscript extensively.

Disadvantages:

1.Insufficient interpretation of the results: in the analysis of the results, the interpretation of some phenomena and conclusions is not deep enough, and there is a lack of discussion on the mechanism behind them. For example, the analysis of the causes for the differences in carbon emission efficiency in different regions is not comprehensive enough, so more in-depth analysis should be conducted from the aspects of economic structure, industrial policy, energy structure and so on, and more targeted suggestions and measures should be put forward.

**Answer:** Thank you for the comment. We agree with your suggestions and we have merged the paragraph with the results analysis section, as can be seen in the section 3, 4 and 5. In addition, we have tried our best to summarize the reasons for the difference in carbon emission reduction efficiency in different regions and analyzed from economic structure, industrial policy, energy structure and other aspects. Hope to get your approval. In addition, more targeted suggestions and measures have been put forward.

For example,

Anhui and northern Jiangsu show a larger increase, especially Anqing, which rises from 0.5308 at the beginning to 0.8872 later. This may be because these latecomer cities, in the stage of new urbanization, timely changed their original extensive land use patterns, abandoned the original concept of promoting economic development with heavy industry as the pillar industry, and carried out corresponding industrial transformation, accelerating the construction of green spaces in new urban areas, thereby reducing carbon emissions under the same energy consumption. (Lines 435-442)

This may be due to the rapid urban expansion stage, where the government converted more and more urban green space into construction land to promote economic development, thus greatly reducing the carbon sequestration capacity of green space. (Lines 451-467)

In the second stage (Figure 7), Anhui Province performed the best in terms of efficiency in the sustainable land use stage from 2011 to 2020, maintaining a stable average of 0.7127 and continuously improving every year. The reason is that due to a certain gap in the total GDP of Anhui compared to Jiangsu, Zhejiang, and Shanghai, Anhui's carbon emissions level is lower than that of Jiangsu, Zhejiang, and Shanghai. The financial investment related to environmental protection is similar among different cities, and the reduction of energy consumption waste and garbage caused by population outflow has led Anhui to invest more in urban green space construction. (Lines 499-533)

In terms of the TCEI of land use calculation, total efficiency has obvious spatial and temporal heterogeneity. It is not unexpected that provincial capitals, metropolitan areas, and coastal developed cities show higher TCEI. This is because as the provincial capitals of their respective provinces, although these cities have a relatively high population size and built-up area, they have already relocated heavy polluting industries and carried out a large amount of urban green space construction in conjunction with the existing suburban green spaces in the city during the urban development process. (Lines 724-753)

2. Writing expression needs to be improved: some sentences in the paper are not accurate or fluent, and need to be further modified and improved. For example, avoid using unprofessional expressions such as "we". It is suggested to improve the writing quality and expression accuracy of the paper.

**Answer:** Thanks for your suggestion. The writing and expression of the paper has been modified and improved by a native English speaker from the USA. And we hope the revised manuscript could be acceptable for you. Moreover, we have tried to change “We” to “This research”, " Meanwhile " to “In addition” or “Moreover” in order to be more professional, as seen in Abstract section. The certification in English editing are as follows.

For example,

**5.1 Conclusions**

This research employs a dynamic two-stage DEA model to build a TCEI assessment framework. Subsequently, the study determines TCEI in the land use process of YRD from 2011 to 2020 and further predict their dynamic evolution. The findings suggest the following. (Lines 785-795)

3. Insufficient interpretation of variables: the data source of each variable is not clearly specified in the paper; the large sample problems required by the deep learning algorithm LSTM were not fully considered, the effect size test was not fully conducted, and the concerns about possible overfitting problems were not effectively solved. It is suggested that the variable meaning and data sources of all equations be explained in detail in the paper to enhance the comprehensibility of the paper.

**Answer:** Thank the reviewer for continuing to point out the details. We think this is an excellent suggestion. We first supplement the effect size test, explain the model overfitting problem, and add explanatory notes on the sources of variables.

1. Supplement on effect size test

To avoid some problems like the size of the effect is not practical or of little value, Cohen (1992) suggested fixing the power at 0.80 (β = 0.20), which is also a convention proposed for general use. The approach of [Dupont and Plummer (1990)](mk:@MSITStore:C:\Program%20Files%20(x86)\PS%20Power%20and%20Sample%20Size\pshelp.chm::/html/pshe23av.htm#ref3) is used for paired and independent samples. The ratio of number of patients in the samples being compared may be specified by the user. This method produces results that are in close agreement with those of [Pearson and Hartley (1970)](mk:@MSITStore:C:\Program%20Files%20(x86)\PS%20Power%20and%20Sample%20Size\pshelp.chm::/html/pshe23av.htm#ref6).

Based on this, this research is planning a study with 44 experimental subjects and 176 control subjects. In addition, the Type I error probability associated with this test of this null hypothesis (α) is 0.1. In a previous study the response within each subject group was normally distributed with standard deviation 0.168. If the true difference in the experimental and control means is 0.071, we will be able to reject the null hypothesis that the population means of the experimental and control groups are equal with probability (power) 0.804. This suggests that our experimental and control data are reasonable. The following is a statistical power graph based on the calculation results.

References:

<http://dx.doi.org/10.20982/tqmp.14.4.p242>.

<https://psycnet.apa.org/doi/10.1037/a0024338>.

<https://psycnet.apa.org/doi/10.1037/0021-9010.90.1.94>.

2. Testing and optimization of overfitting problems

First of all, the fitting results of our four variables are good, and the adjusted R^2^ is at the level of 0.9. Secondly, we use the learning curve to determine whether there is overfitting phenomenon in the prediction of efficiency value. As shown in the figure, y (efficiency) training set and verification set both have lower loss errors. On the one hand, the training set loss gradually decreases and flattens with the increase of training samples, indicating that adding more training samples does not improve the performance of the model on training data. On the other hand, the learning curve of the training set has a high validation loss at the beginning, and gradually decreases and tends to be flat with the increase of training samples. Therefore, it is judged that there is no overfitting phenomenon in the prediction of the efficiency value y(efficiency).

Figure 1. Plot of Input Variable Fit.

Figure 2. Graph of training set and test set learning curve

In addition, in view of the possibility of slight fitting in small samples, this research adopted regularization and random silencing of some neurons during each training to reduce the complexity of the model and the degree of dependence between neurons, so as to enhance the generalization ability of the model.

References:

Ying, X. (2019, February). An overview of overfitting and its solutions. In Journal of physics: Conference series (Vol. 1168, p. 022022). IOP Publishing.

Hawkins, D. M. (2004). The problem of overfitting. Journal of chemical information and computer sciences, 44(1), 1-12.

Vul, E., Goodman, N., Griffiths, T. L., & Tenenbaum, J. B. (2014). One and done? Optimal decisions from very few samples. Cognitive science, 38(4), 599-637.

3. Supplement on the sources of variables

We have supplemented the data sources and the meaning of variables in the table, as can be seen in Appendix A. (Descriptions of input and output variables)

(Line 1028-1029)
